# Supplementary material for: Impact of COVID-19 Pandemic on Adherence to Chronic Therapies: A Systematic Review
Source: Int J Environ Res Public Health. 2023 Feb 21;20(5):3825. doi: 10.3390/ijerph20053825 (PMC10001666; doi:10.3390/ijerph20053825)
Supplement: Supplementary file 1 [file ijerph-20-03825-s001.zip › ijerph-2206535-supplementary.pdf]

SUPPLEMENTARY MATERIAL

Impact of COVID-19 Pandemic on Adherence to Chronic Therapies: A Systematic Review

Elena Olmastroni [1,*], Federica Galimberti [2], Elena Tragni [1], Alberico L. Catapano [1,2] and Manuela Casula [1,2]

[1] Epidemiology and Preventive Pharmacology Service (SEFAP), Department of Pharmacological and Biomolecular Sciences, University of Milan, 20133 Milan, Italy
[2] IRCCS MultiMedica, 20099 Sesto San Giovanni (MI), Italy
* Correspondence: elena.olmastroni@unimi.it

Supplemental methods

Literature searches

PubMed

("medication adherence"[MeSH Terms] OR ("medication"[All Fields] AND "adherence"[All Fields]) OR "medication adherence"[All Fields] OR ("medication"[All Fields] AND "compliance"[All Fields]) OR "medication compliance"[All Fields] OR ("treatment adherence and compliance"[MeSH Terms] OR ("treatment"[All Fields] AND "adherence"[All Fields] AND "compliance"[All Fields]) OR "treatment adherence and compliance"[All Fields] OR ("treatment"[All Fields] AND "adherence"[All Fields]) OR "treatment adherence"[All Fields])) AND ("sars cov 2"[MeSH Terms] OR "sars cov 2"[All Fields] OR "covid"[All Fields] OR "covid 19"[MeSH Terms] OR "covid 19"[All Fields] OR ("pandemic s"[All Fields] OR "pandemically"[All Fields] OR "pandemicity"[All Fields] OR "pandemics"[MeSH Terms] OR "pandemics"[All Fields] OR "pandemic"[All Fields]))

Embase

('medication compliance'/exp OR 'medication compliance' OR 'medication adherence assessment'/exp OR 'medication adherence assessment' OR 'patient compliance'/exp OR 'patient compliance' OR 'medication adherence'/exp OR 'medication adherence' OR (('medication'/exp OR medication) AND ('adherence'/exp OR adherence))) AND ('severe acute respiratory syndrome coronavirus 2'/exp OR 'severe acute respiratory syndrome coronavirus 2' OR 'coronavirus disease 2019'/exp OR 'coronavirus disease 2019' OR (('pandemic'/exp OR 'pandemic') AND ('covid 19'/exp OR 'covid 19')))

Web of Science

("medication adherence" OR ("medication" AND "adherence") OR "medication adherence" OR ("medication" AND "compliance") OR "medication compliance" OR ("treatment adherence and compliance" OR ("treatment" AND "adherence" AND "compliance") OR "treatment adherence and compliance" OR ("treatment" AND "adherence") OR "treatment adherence")) AND ("sars cov 2" OR "sars cov 2" OR "covid" OR "covid 19" OR "covid 19" OR ("pandemic s" OR "pandemically" OR "pandemicity" OR "pandemics" OR "pandemics" OR "pandemic"))

Supplementary Table S1. Quality assessment of studies (n = 12) included in the primary outcome evaluation using the National Institutes of Health (NIH) quality assessment tool for observational cohorts and cross-sectional studies.

**Major Components**	**Kahn N [1]**	**Kaye L [2]**	**Hasseli R [3]**	**Wagner Z [4]**	**De Vincentis S [5]**	**Dhruve H [6]**	**Uchida H [7]**	**Garg R [8]**	**Racette L [9]**	**Ramey OL [10]**	**Romagnoli A [11]**	**Villalobos Violán V [12]**
1. Was the research question or **objective** in this paper clearly stated?	yes	yes	yes	yes	yes	yes	yes	yes	yes	yes	yes	yes
2. Was the **study population** clearly specified and defined?	yes	no	yes	yes	yes	yes	no	yes	yes	yes	yes	yes
3. Was the **participation rate** of eligible persons at least 50%?	yes	nr	nr	yes	yes	yes	nr	yes	yes	yes	yes	nr
4. Were all the subjects selected or recruited from the **same or similar populations** (including the same time period)? Were inclusion and exclusion criteria for being in the study prespecified and applied uniformly to all participants?	yes	yes	yes	yes	yes	yes	yes	yes	yes	yes	yes	yes
5. Was a **sample size** justification, power description, **or variance and effect estimates** provided?	no	no	no	no	no	no	no	no	no	no	no	no
6. For the analyses in this paper, **were the exposure(s) of interest measured prior to the outcome(s) being measured**?	yes	yes	yes	yes	yes	yes	yes	yes	yes	yes	yes	yes
7. Was the **timeframe sufficient** so that one could reasonably expect to see an association between exposure and outcome if it existed?	yes	yes	yes	yes	yes	yes	yes	yes	yes	yes	yes	yes
8. For exposures that can vary in amount or level, did the study examine **different levels of the exposure** as related to the outcome (e.g., categories of exposure, or exposure measured as continuous variable)?	na	na	na	na	na	na	na	na	na	na	na	na
9. Were the **exposure measures** (independent variables) clearly defined, valid, reliable, and implemented consistently across all study participants?	yes	yes	yes	yes	yes	yes	yes	yes	yes	yes	yes	yes
10. Was the **exposure(s)** assessed more than once over time?	na	na	na	na	na	na	na	na	na	na	na	na
11. Were the o**utcome measures (dependent variables) clearly defined**, valid, reliable, and	yes	no	no	yes	yes	yes	yes	yes	yes	yes	yes	yes

implemented consistently across all study participants?												
12. Were the **outcome assessors blinded** to the exposure status of participants?	no	no	no	no	no	no	no	no	no	no	no	no
13. Was **loss to follow-up** after baseline 20% or less?	yes	nr	na	yes	yes	yes	nr	nr	nr	nr	nr	na
14. Were **key potential confounding variables** measured and adjusted statistically for their impact on the relationship between exposure(s) and outcome(s)?	no	no	no	yes	no	no	no	no	no	no	no	no

na: not applicable; nr: not reported.

References for Supplementary Table 1

1. Khan N, Patel D, Xie D, Pernes T, Lewis J, Yang YX. Adherence of Infusible Biologics During the Time of COVID-19 Among Patients With Inflammatory Bowel Disease: A Nationwide Veterans Affairs Cohort Study. Gastroenterology. 2020;159(4):1592-4 e1.
2. Kaye L, Theye B, Smeenk I, Gondalia R, Barrett MA, Stempel DA. Changes in medication adherence among patients with asthma and COPD during the COVID-19 pandemic. J Allergy Clin Immunol Pract. 2020;8(7):2384-5.
3. Hasseli R, Muller-Ladner U, Keil F, Broll M, Dormann A, Frabel C, et al. The influence of the SARS-CoV-2 lockdown on patients with inflammatory rheumatic diseases on their adherence to immunomodulatory medication: a cross sectional study over 3 months in Germany. Rheumatology (Oxford). 2021;60(SI):SI51-SI8.
4. Wagner Z, Mukasa B, Nakakande J, Stecher C, Saya U, Linnemayr S. Impact of the COVID-19 Pandemic on Use of HIV Care, Antiretroviral Therapy Adherence, and Viral Suppression: An Observational Cohort Study From Uganda. J Acquir Immune Defic Syndr. 2021;88(5):448-56.
5. De Vincentis S, Domenici D, Ansaloni A, Boselli G, D'Angelo G, Russo A, et al. COVID-19 lockdown negatively impacted on adherence to denosumab therapy: incidence of non-traumatic fractures and role of telemedicine. J Endocrinol Invest. 2022.
6. Dhruve H, d'Ancona G, Holmes S, Dhariwal J, Nanzer AM, Jackson DJ. Prescribing Patterns and Treatment Adherence in Patients with Asthma During the COVID-19 Pandemic. J Allergy Clin Immunol Pract. 2022;10(1):100-7 e2.
7. Uchida H, Kamata M, Egawa S, Nagata M, Fukaya S, Hayashi K, et al. Impact of the COVID-19 pandemic on biologic treatment in psoriasis patients: A single-center retrospective study in Japan. J Dermatol. 2022;49(6):624-8.
8. Garg R, Kitchen SA, Men S, Campbell TJ, Bozinoff N, Tadrous M, et al. Impact of the COVID-19 pandemic on the prevalence of opioid agonist therapy discontinuation in Ontario, Canada: A population-based time series analysis. Drug Alcohol De-pend. 2022;236:109459.
9. Racette L, Abu SL, Poleon S, Thomas T, Sabbagh N, Girkin CA. The Impact of the Coronavirus Disease 2019 Pandemic on Adherence to Ocular Hypotensive Medication in Patients with Primary Open-Angle Glaucoma. Ophthalmology. 2022;129(3):258-66.
10. Ramey OL, Silva Almodovar A, Nahata MC. Medication adherence in Medicare-enrolled older adults with asthma before and during the coronavirus disease 2019 pandemic. Ann Allergy Asthma Immunol. 2022;128(5):561-7 e1.
11. Romagnoli A, Santoleri F, Costantini A. The impact of COVID-19 on chronic therapies: the Pescara (ASL) local health authority experience in Italy. Curr Med Res Opin. 2022;38(2):311-6.
12. Villalobos Violan V, Gandolfo Cano MDM, Vicente EM, Trujillo MJT, Gonzalez Mancebo E. Influence of the COVID-19 pandemic on the prescription and adherence to allergen-specific immunotherapy. Clin Exp Allergy. 2022;52(7):916-7.
